# Short-Term HRV Detection and Human Fatigue State Analysis Based on Optical Fiber Sensing Technology

**DOI:** 10.3390/s22186940

**Published:** 2022-09-14

**Authors:** Siqi Hu, Huaguan Lin, Quanqing Zhang, Sheng Wang, Qingbing Zeng, Sailing He

**Affiliations:** 1 Shanghai Aviation Electric Co., Ltd., Shanghai 201100, China; 2 College of optical science and engineering, Zhejiang University, Hangzhou 310000, China

**Keywords:** mental fatigue, optical fiber sensing, FBG

## Abstract

Mental fatigue is a key cause of chronic diseases and traffic accidents, which is difficult to be quantitatively evaluated. In order to non-intrusively detect fatigue state, an optical fiber sensing system is proposed, which is non-invasive and does not require direct contact with skin. The fiber sensor was fabricated through phase mask exposure method and packaged by sensitivity-enhanced structure, which can suppress transverse force and increase signal amplitude by 5%. A fatigue-inducing experiment was carried out, and the heartbeat signals of 20 subjects under different fatigue states were collected by the proposed sensing system. A series of heart rate variability indicators were calculated from the sensing signals, and their statistical significance for fatigue was analyzed. The experiment results showed that the values of SDNN and LF/HF increased significantly with subjects’ fatigue level. This study shows that the proposed fiber optic sensing system has practical value in fatigue state monitoring.

## 1. Introduction

Studies have shown that both stroke and Karoshi have a strong relationship with mental fatigue caused by overwork, and high-intensity work will increase the incidence of cardiovascular disease [1,2]. Apart from the damage to physical health, mental fatigue has negative effects on memory, judgment, decision-making, and emotional management [3,4,5]. Long-term overwork can lead to stress and tension, and further lead to higher accident rates and lower production efficiency. Therefore, the development of intelligent wearable devices for real-time monitoring of human fatigue status is of great significance, especially in driving, medical care, scientific research, military and other work scenarios.

It has been confirmed that the mechanism of fatigue is related to autonomic nerve activity [6]. In 1996, a special committee composed of the European Society of Cardiology and the North American Society of Pacing and Electrophysiology clearly pointed out that heart rate variability (HRV) is the best quantitative indicator for evaluating autonomic activity [7]. So HRV can reflect a person’s mental fatigue state. HRV is usually extracted from ECG signal and can change with different activities. HRV refers to the slight difference in adjacent heartbeat interval, which is determined by the length of two adjacent R wave peaks of electrocardiogram (ECG) signal (R-R intervals). More specifically, HRV’s time-domain, frequency-domain indicators and nonlinear scatter plot information have important clinical guiding value for the prediction of mental fatigue [4,5,6,8,9,10,11,12,13]. A number of research projects on driver fatigue were conducted. Li found that spectral analysis of HRV can reflect the balance of sympathetic and parasympathetic activity and so be used as a quantitative indicator of driver mental fatigue [8]. In addition, Patel’s paper presents artificial intelligence-based analysis of HRV can detect early onset of fatigue in drivers, which showed that the LF/HF ratio was significantly higher in fatigue state (*p* = 0.01) [10]. Escorihuela researched the diagnosis of fatigue severity in individuals with chronic fatigue syndrome or myalgic encephalomyelitis (CFS/ME), which showed that CFS/ME patients have decreased RR intervals (*p* < 0.01) and higher LF/HF ratio than in the healthy controls [9].

However, in terms of HRV acquisition methods, in many scenarios, ECG collection can often affect normal working activities. Therefore, it is necessary to invent more convenient wearable monitoring devices to obtain HRV of workers in real time. HRV can be obtained not only from the ECG, but also from the ballistocardiogram (BCG) and the pulse signal obtained by plethysmography (PPG) technology [14]. In addition, the BCG signal has a good corresponding relationship with the ECG and the PPG signal [14,15,16]. However, ECG and PPG signals are usually measured through direct contact with human skin, while the BCG signal can be easily obtained by non-direct contact method, that is by sensing the micro-strain of the human body [15].

It is well known that optical fiber sensing technology has significant advantages in measuring micro-strain [17,18,19], such as real-time, lightweight, self-organized network, anti-electromagnetic interference, high sensitivity and accuracy. Other commonly used strain detection methods include capacitive-resistive sensors, piezoelectric ceramics and piezoelectric thin films and recording electrical signals [20,21,22]. However, there is a complex magnetic field environment in the high-altitude flight environment, and electronic devices are easily affected by the magnetic fields, and the signal quality is poor. The optical fiber sensing system is a detection system based on all-optical signals, which is resistant to electromagnetic interference and can be embedded in clothing or straps for pilots to wear. In addition, the electronic sensor has a single function, and the optical fiber sensing system has a flexible multiplexing network architecture, which can provide a reserved interface for the follow-up pilot multi-parameter integrated physiological monitor. Therefore, we considered to acquire HRV information through BCG signal obtained by an optical fiber sensor.

## 2. Materials and Methods

### 2.1. Fiber Sensor Fabrication and Packaging

As mentioned above, the basic principle of detecting human physiological parameters by means of optical fiber technology is to sense the micro-strain produced by physiological activities, such as respiration and heartbeat. So, the optical fiber sensor needed here is one that has high strain sensitivity. On the type selection of the fiber optic sensor, considering the commercial value of fiber Bragg grating (FBG), including but not limited to high technical maturity and the ability of commercial mass production, in order to improve the practicability of the developed device, here we use FBG to collect physiological parameters. Because FBG has the advantages of small size, low splicing loss, full compatibility with optical fibers and can be embedded in smart materials, and its resonant wavelength is sensitive to changes in external physical quantities such as temperature, strain, refractive index and gas concentration, it has been widely used in the production of fiber lasers and optical fiber sensing devices.

**Figure 1:**
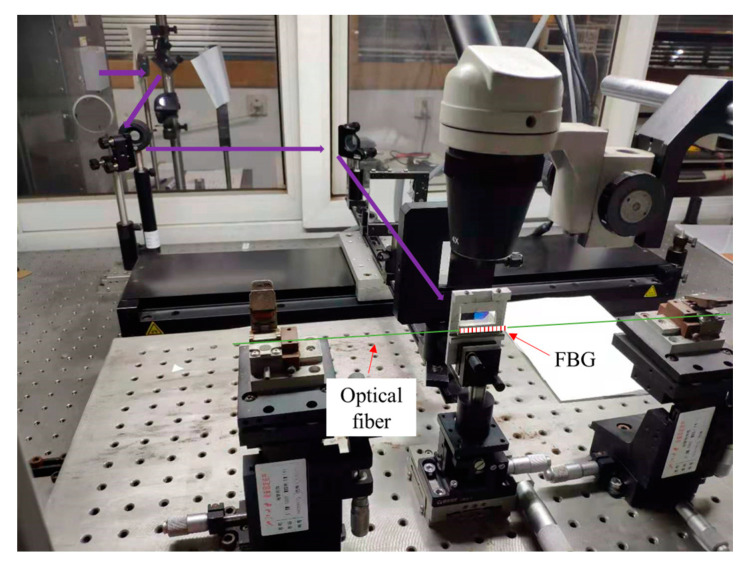
The phase-mask-UV-exposure FBG fabrication system.

FBG can be obtained by means such as double-beam interference, phase mask exposure and femtosecond laser writing. Likewise, considering cost and industrial feasibility, we choose the phase mask method [23], which is currently the only commercial production method for FBG. The UV mask exposure system built in the laboratory is shown in Figure 1.

The phase mask used in the FBG fabrication method was produced by holographic interference. During the FBG fabrication process, the phase mask is placed in front of the photosensitive fiber (IXF-PHO-CMF, IXBlue, Besançon, France). In addition, a 248 nm UV light source passes through a beam shaping optical path and is diffracted to the optical fiber through the phase mask to form periodic interference fringes on the fiber, which modulate the core refractive index of the fiber periodically and produce FBG. This grating-making method does not depend on the wavelength of the incident light, but is only related to the period of the phase mask. Therefore, the high coherence of the light source is not demanding, which simplifies the manufacturing system of FBG. This fabrication method with a low-coherence light source and a phase mask is very convenient and important, because various positions and structures of gratings can be achieved by precisely adjusting the phase mask and the UV exposure technique. It greatly simplifies the fabrication process of fiber gratings. In addition, it is the only commercial method for fabricating fiber gratings in large quantities at present [24].

**Figure 2:**
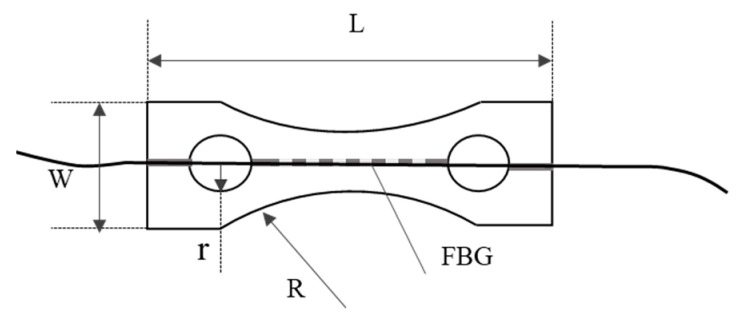
The schematic diagram of FBG package structure.

**Figure 3:**
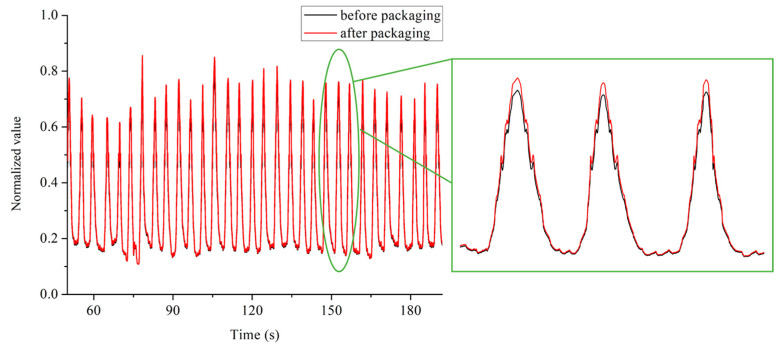
Signal diagrams before and after FBG packaging.

The packaging structure can not only protect the optical fiber sensor, but also enhance the sensitivity of the strain sensing performance. Through proper design of the packaging structure, the sensitivity can be enhanced by suppressing or compensating the sensing noise and reducing strain conducting loss of the sensor [19,25,26]. Here the dimensions of the package substrate are length L = 55 mm, width W = 16 mm, small circle radius r = 4 mm, arc radius R = 30 mm, groove depth 1 mm, using composite materials (70% nylon and 30% E-glass fiber of 17 μm diameter combining composite), elastic modulus E ≈ 1800 MPa. This kind of composite material makes it easy to create high-precision small pores, thin walls, rod handles and gears and other complex parts or lattice structures. It has the good flexibility of nylon and the good strength of glass fiber, which is very suitable for packaging tiny fiber grating sensors. Figure 2 is the designed structure of the package substrate. We embedded the bare and packaged FBGs into the strap in parallel, and simultaneously acquired the two channel signals with the demodulator. Comparing the signals collected by the FBG before and after packaging, it can be measured that the strain sensitivity after encapsulation is increased by about 5%, as shown in Figure 3. The collected original signal is not strictly periodic in the time domain. The large envelopes are the contraction and expansion of the thoracic cavity caused by breathing, resulting in large and low-frequency changes in the center wavelength reflected by the FBG sensor. The burrs accompanying the envelopes are actually the effective signal caused by the heartbeat activity with small amplitude and higher frequency, and it is also the data source for subsequent HRV calculation.

### 2.2. Principle of the Optical Fiber Sensing System

**Figure 4:**
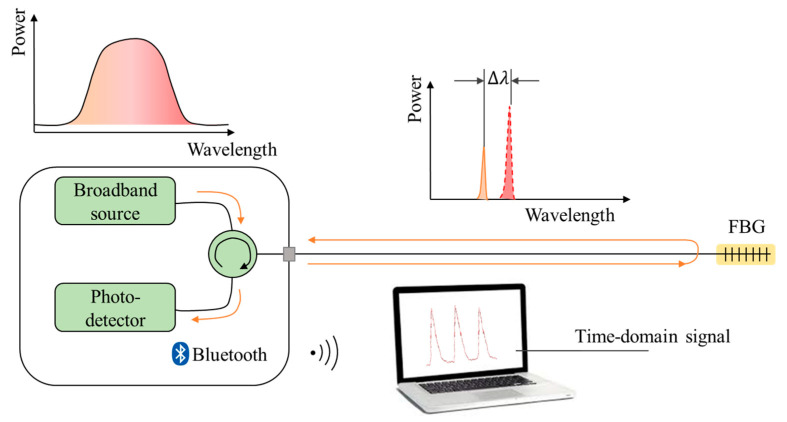
The schematic diagram of the optical fiber sensing system.

The schematic diagram of the proposed sensing system is shown in Figure 4. A broad band light source was launched to FBG through the leading fiber, while the real-time reflecting signal from FBG was recorded by the demodulator and then analyzed by computer program.

The light source, FBG and the demodulator are connected through a circulator, and the demodulator transmits information to the computer through Bluetooth communication. The breathing and heartbeat activities of the human body cause the expansion and contraction of the thoracic cavity, which imposes strain on the attached FBG. In addition, the period Λ of FBG changes accordingly. The center wavelength reflected by FBG according to Bragg condition:

(1) $$\lambda_{B}=2n_{eff}\Lambda$$

shifts with its period Λ, in which $\lambda_{B}$ is the center wavelength under Bragg condition and $n_{eff}$ is the effective refractive index of FBG.

**Figure 5:**
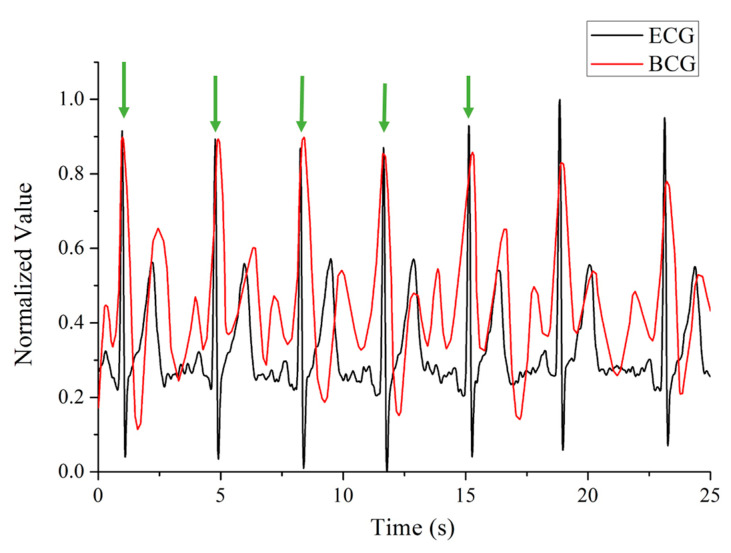
Comparison of the measured BCG and ECG signals.

**Figure 6:**
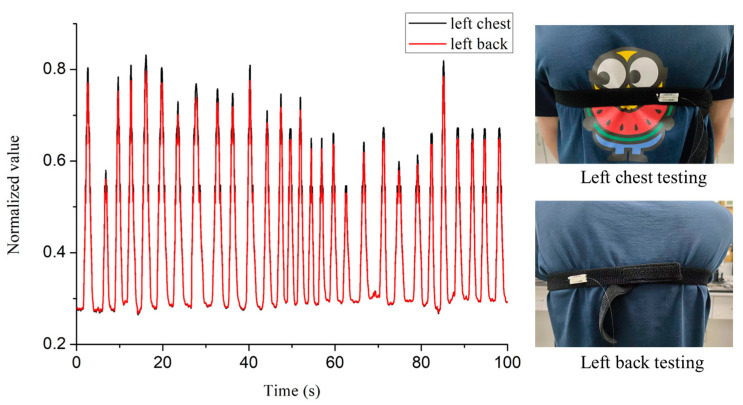
The signal results of the front and back of the left chest testing.

During the test, the FBG was bound on the subject’s chest, close to heart, and the demodulator recorded the time-domain signal returned from the FBG in real time. The original signal contains both the respiration and heartbeat components, which are separated later by post data processing. The heartbeat signal is calibrated by the ECG device, shown in Figure 5. It is found that FBG has ideal signal response on both the left chest and back positions, as shown in Figure 6. In order to explore the application potential of the system in personnel status recognition, 10 male and 10 female subjects were invited for testing experiment. We collected their respiration and heartbeats for 5 min using optical fiber system before and after doing strenuous exercise respectively. The vigorous exercise in the experimental setup was high leg raises for 5 min.

## 3. Results

### 3.1. Experimental Process

Twenty healthy college students with no history of heart disease participated in the experiment, the ratio of male to female was 1:1, and their age range was 25 ± 4 years old. All people without heart disease are selected here in order to exclude the interference of other factors, and try to make the difference in HRV index only related to fatigue state. Studies have shown that the HRV index of heart disease patients is lower than that of normal people [9], so different objective samples may introduce errors or abnormal data when carrying out statistical analysis. When performing fatigue discrimination based on HRV indicators for other special groups such as patients with heart disease, the results need to be revised.

In the experiment, the Checkme Lite ECG recording device of Lepu Medical was used as the calibration equipment for the heartbeat signal, and the respiration signal was calibrated by counting observation method. The experiment research was carried out at a room temperature of 27 °C and a relative humidity of 65%, and each subject participated in the experimental test for about 105 min. During the test, the packaged FBG sensor was fixed on the chest of the male and the back of the female subject through a strap, where was as close as to the heart position.

**Figure 7:**
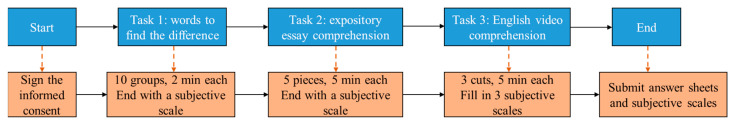
Fatigue monitoring experiment process.

During test, the subjects were asked to complete different types of questions in turn. Each subject completed 3 tasks, which spent 105 min in total. Task 1 is 10 groups of words to find the difference, 2 min for each group (20 min in total). Task 2 was 5 groups of expository essay-reading comprehension, 5 min each (25 min in total). In addition, task 3 was to watch 3 English videos and answer questions about the video content, each 20 min (60 min in total). The experimental process is shown in Figure 7. During the test, at the end of task 1, task 2 and after answering each video’s questions, the subject was asked to fill in the subjective fatigue scale. The fatigue scale was able to divide the subjects into 3 levels of fatigue state according to their score. In addition, marking the time when the subjects fill in the subjective scale, which is convenient to identify the physiological data corresponding to different fatigue states.

### 3.2. Data Processing Method

**Figure 8:**
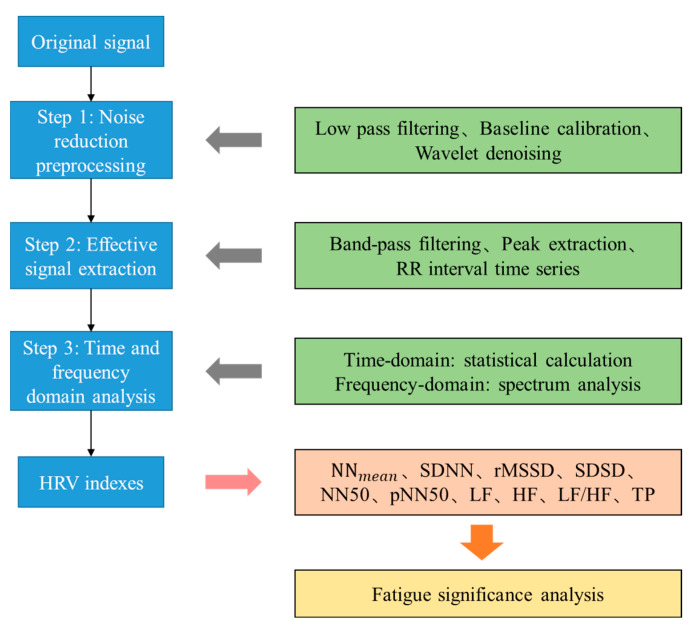
Signal processing flowchart.

The signal processing process mainly includes three steps: noise reduction, useful signal extraction and time-frequency analysis, as shown in Figure 8, which is the signal processing flow. The original signal collected by optical fiber sensing equipment is inevitably affected by high-frequency noise, baseline drift, motion artifacts and other noises. These noises are removed according to low-pass filtering, baseline fluctuation trend and data cycle repeatability, respectively.

**Figure 9:**
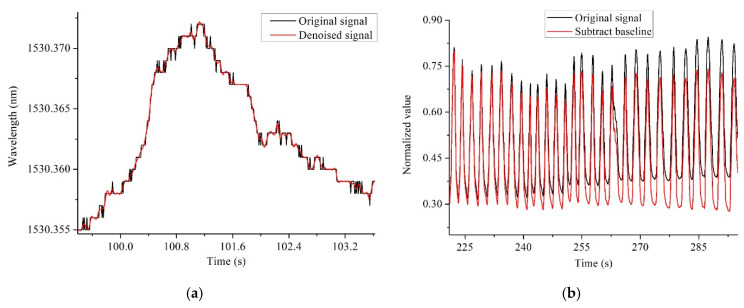
(**a**) Before and after signal noise reduction; (**b**) Before and after the elimination of signal baseline drift.

**Figure 10:**
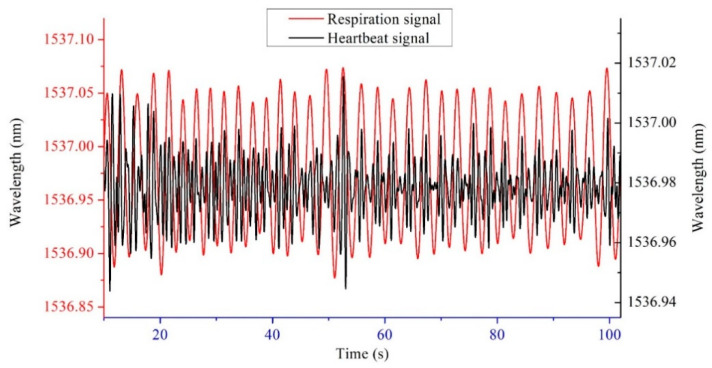
Respiration and heartbeat signal separation diagram.

**Figure 11:**
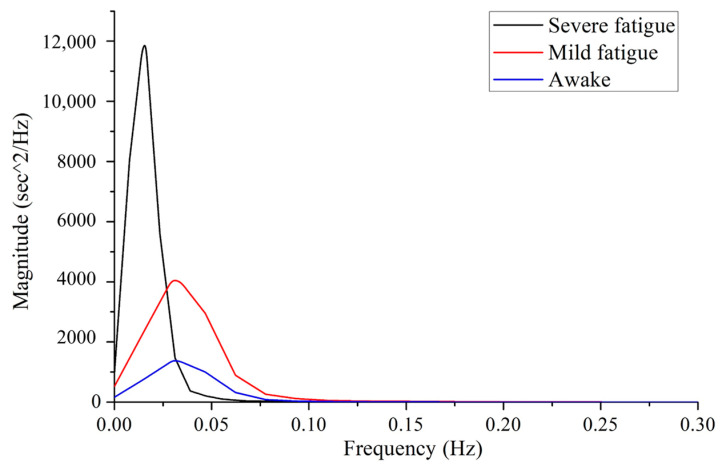
The spectrum of the heartbeat signal under different fatigue states.

The original signal and the denoised signal are shown in Figure 9. The sensor is close to human body and so radiated by human body temperature. The elevated temperature causes the center wavelength reflected by FBG to shift, resulting in a slow baseline shift of the signal. Then the heartbeat signal can be separated from the original. According to the frequency difference of mechanical vibration caused by respiration and heartbeat, the respiration signal was filtered out by band-pass filtering of 1~35 Hz. The baseline drift was also eliminated in this process. For large-scale abnormal body movements, threshold noise reduction method was used to eliminate them. Then decompose the remaining signal with wavelet transform, and identify the R-peak sequence of BCG signal with ECG signal alignment. Figure 10 shows the respiration and heartbeat signal separated from the original. Finally, the time and frequency index calculation is performed on the heartbeat signal. The calculation method of the time domain indexes is to compare the BCG signal to the ECG signal collected by Checkme Lite, use wavelet transform and peak extraction to obtain the R peak sequence of the BCG signal, and further calculate the RR interval sequence, that is, the HRV sequence. The considered time domain indexes include NN_mean (mean value of RR intervals), SDNN (standard deviation of RR intervals), rMSSD (root mean square of differences between adjacent RR intervals), SDSD (standard deviation of the differences between adjacent RR intervals), NN50 (the number of differences of adjacent RR intervals that are greater than 50 ms) and pNN50 (the percentage of NN50 in the total). The calculation method of the frequency domain indexes is usually to interpolate the RR interval to make it an equidistant sampling sequence, and then perform a fast Fourier transform (FFT) on it to obtain its frequency estimation spectrum, such as Figure 11. Finally, the frequency domain indexes of HRV can be obtained through spectrum analysis, including LF (low frequency power, 0.04~0.15 Hz), HF (high frequency power, 0.15~0.4 Hz), LF/HF (the ratio of low frequency power to high frequency power), and TP (total power). It is worth noting that LF plus HF does not equal TP, as the spectrum also contains other components such as VLF (very low frequency power).

### 3.3. Data Analysis

**Figure 12:**
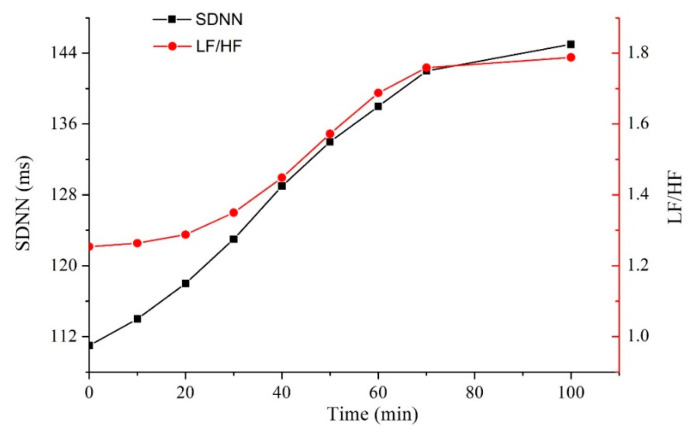
Polylines of SDNN and LF/HF of the subjects over time.

In order to figure out the eigenvalues which have stronger correlation with fatigue, the obtained 10 HRV indexes are sorted, selected and analyzed by SPSS 20.0 software (IBM, Armonk, NY, USA). The statistical contents include the sample number, average value and standard error of HRV parameters corresponding to different fatigue degrees. ANOVA (one-way analysis of variance) was used to analyze the differences between HRV indexes and different fatigue levels, with *p* < 0.05 representing a significant difference [27,28]. The characteristic indexes that can effectively discriminate the fatigue state are selected. Table 1 shows the common HRV time domain and frequency domain indexes. RRi refers to the time interval between adjacent R peaks and NNi is the difference between adjacent RR intervals. Table 2 shows the statistical analysis results of the three states of fatigue (awake, mild fatigue and severe fatigue (drowsiness)) and HRV indexes, with data recorded in the form of x ± s. It can be found that the SDNN, SDSD, LF, HF and LF/HF indexes were statistically significant (*p* < 0.05) in different fatigue degrees, while RR_mean, rMSSD and pNN50 were not statistically significant (*p* > 0.05). Among the significant ones, SDNN and LF/HF have the smallest *p* values and so are the most correlated indexes. The experimental results show that with the deepening of the fatigue degree, the SDNN and LF/HF of the objects both increased significantly. The results are shown in Figure 12.

## 4. Conclusions and Discussion

A heartbeat BCG signal acquisition system based on optical fiber sensing technology was developed in this paper. The sensing system is implemented based on an all-optical network, which can well avoid high-altitude electromagnetic interference. It is small in size, light in weight, non-invasive and wearable. It is reserved interface for future pilots’ core body temperature, blood oxygen, blood pressure and other physiological parameters’ monitoring. In addition, the feasibility of the system in identifying human fatigue status is studied. The experiment showed that the optical fiber sensor could accurately obtain BCG signal, from which HRV indexes needed for fatigue evaluation could be extracted. In addition, in terms of fatigue state identification, the statistical analysis results showed that the SDNN, SDSD, LF, HF and LF/HF indexes of HRV have significant (*p* < 0.05) correlation with fatigue. Among them, SDNN and LF/HF were most relevant with fatigue status. The results revealed the values of SDNN and LF/HF both increased obviously with the deepening of fatigue.

It is of great practical significance to study a new and more convenient way to the identify fatigue state in real time. Excessive fatigue can affect the normal thinking and work efficiency, which can cause serious consequences in some scenarios. In this paper, the experimental research and result analysis verify that the BCG signal measured by the optical fiber sensor has the same research and analysis value as the ECG signal, enriches the way of obtaining HRV indexes, and explores the relationship between the HRV indexes and mental fatigue. It lays the foundation for further research on real-time mental fatigue identification and helps to manage the performance tasks properly in the future. In addition, by embedding the fiber into mattresses, cushions and other items, unbundled and non-inductive monitoring of physical parameters can be achieved in scenarios such as hospitals, nursing homes, and families. It is expected to be widely used in smart medical and smart home industries in the future. The developed optical fiber sensing system can meet the large demand for long-term observation of chronic diseases brought about by the aging of the population, which can alleviate the run on medical resources.

## Figures and Tables

**Table 1:** Definition and formula of HRV indexes.

Type	Index	Definition	Formula (Unit)
Time-domain	${\mathrm{RR}}_{mean}$	mean value of RR intervals	$\frac{\sum_{i=1}^{N}\left(RR_{i}\right)}{N}$ (ms)
	SDNN	standard deviation of RR intervals	sqrt $\left(\frac{\sum_{i=1}^{N}{\left(RR_{i}-mRR\right)}^{2}}{N-1}\right)$ (ms)
	rMSSD	root mean square of differences between adjacent RR intervals	$sqrt\left(mean\left({\left(RR_{i+1}-RR_{i}\right)}^{2}\right)\right)\;\left(\mathrm{ms}\right)$
	SDSD	standard deviation of the differences between adjacent RR intervals	sqrt $\left(\frac{\sum_{i=1}^{N}{\left(NN_{i}-mNN\right)}^{2}}{N-1}\right)$ (ms)
	pNN50	the percentage of the number of NN interval > 50 ms in the total	$\frac{count\left(|NN_{i+1}-NN_{i}|\right)>50\;\mathrm{ms}\;}{N-1}\times 100\%$
Frequency-domain	LF	low frequency power, 0.04~0.15 Hz	FFT and Integration
	HF	high frequency power, 0.15~0.4 Hz	
	LF/HF	the ratio of low frequency power to high frequency power	

**Table 2:** Significant statistical results of HRV indexes.

Type	Index	Awake	Mild Fatigue	Severe Fatigue	*p* Value
Time-domain	${\mathrm{RR}}_{mean}$	786.07 ± 30.92	883.49 ± 36.21	866.08 ± 57.26	0.102
	SDNN	111.05 ± 7.36	132.61 ± 6.42	146.01 ± 5.72	0.003 ^1^
	rMSSD	31.25 ± 2.07	34.68 ± 3.13	32.09 ± 2.40	0.472
	SDSD	27.21 ± 5.58	30.72 ± 7.25	39.51 ± 6.60	0.034 ^1^
	pNN50	10.63 ± 3.17	13.35 ± 2.40	12.57 ± 5.02	0.483
Frequency-domain	LF	1454.93 ± 36.81	1541.32 ± 42.27	1630.63 ± 56.72	0.024 ^1^
	HF	1167.92 ± 40.80	1063.28 ± 44.01	912.32 ± 37.28	0.035 ^1^
	LF/HF	1.25 ± 1.31	1.57 ± 1.02	1.78 ± 1.42	0.001 ^1^

^1^ *p* < 0.05, statistically significant.

## Data Availability

Not applicable.
